# Synchrotron microtomography of a *Nothosaurus marchicus* skull informs on nothosaurian physiology and neurosensory adaptations in early Sauropterygia

**DOI:** 10.1371/journal.pone.0188509

**Published:** 2018-01-03

**Authors:** Dennis F. A. E. Voeten, Tobias Reich, Ricardo Araújo, Torsten M. Scheyer

**Affiliations:** 1 European Synchrotron Radiation Facility, Grenoble, France; 2 Department of Zoology and Laboratory of Ornithology, Palacký University, Olomouc, Czech Republic; 3 University of Zurich, Palaeontological Institute and Museum, Zurich, Switzerland; 4 Institute for Plasma Research and Nuclear Fusion, Technical University of Lisbon, Lisbon, Portugal; 5 Museum für Naturkunde, Leibniz-Institut für Evolutions- und Biodiversitätsforschung, Berlin, Germany; 6 Institute of Evolutionary Sciences, University of Montpellier 2, Montpellier, France; Indiana University Bloomington, UNITED STATES

## Abstract

Nothosaurs form a subclade of the secondarily marine Sauropterygia that was well represented in late Early to early Late Triassic marine ecosystems. Here we present and discuss the internal skull anatomy of the small piscivorous nothosaur *Nothosaurus marchicus* from coastal to shallow marine Lower Muschelkalk deposits (Anisian) of Winterswijk, The Netherlands, which represents the oldest sauropterygian endocast visualized to date. The cranial endocast is only partially encapsulated by ossified braincase elements. Cranial flattening and lateral constriction by hypertrophied temporal musculature grant the brain a straight, tubular geometry that lacks particularly well-developed cerebral lobes but does potentially involve distinguishable optic lobes, suggesting vision may have represented an important sense during life. Despite large orbit size, the circuitous muscular pathway linking the basisphenoidal and orbital regions indicates poor oculomotor performance. This suggests a rather fixed ocular orientation, although eye placement and neck manoeuvrability could have enabled binocular if not stereoscopic vision. The proportionally large dorsal projection of the braincase endocast towards the well-developed pineal foramen advocates substantial dependence on the corresponding pineal system *in vivo*. Structures corroborating keen olfactory or acoustic senses were not identified. The likely atrophied vomeronasal organ argues against the presence of a forked tongue in *Nothosaurus*, and the relative positioning of external and internal nares contrasts respiratory configurations proposed for pistosauroid sauropterygians. The antorbital domain furthermore accommodates a putative rostral sensory plexus and pronounced lateral nasal glands that were likely exapted as salt glands. Previously proposed nothosaurian ‘foramina eustachii’ arose from architectural constraints on braincase development rather than representing functional foramina. Several modifications to brain shape and accessory organs were achieved through heterochronic development of the cranium, particularly the braincase. In summary, the cranium of *Nothosaurus marchicus* reflects important physiological and neurosensory adaptations that enabled the group’s explosive invasion of shallow marine habitats in the late Early Triassic.

## Introduction

The Permian-Triassic (P-T) mass extinction profoundly influenced the evolutionary history of most taxa that survived the P-T event [1, 2]. Sauropterygia are a particularly successful group of secondarily marine sauropsids of which the oldest recognized fossils date back to the Spathian sub-stage of the Olenekian [3, 4], about 5 million years after the P-T mass extinction [1]. These initially small- to medium-bodied predators exhibited rapid dispersal in the newly formed epicontinental Muschelkalk Sea and along the shallow marine margins of the Tethyan realm [5] in a highly competitive arena shared with a diverse variety of other secondarily marine reptile taxa [1]. Lower Muschelkalk deposits have yielded a suite of related but morphologically distinct families [6], which demonstrates that the Sauropterygia were already established in close to all the higher trophic levels of the food chain by then [1, 7]. Following this initial radiation, several sauropterygian taxa display intrageneric niche partitioning and speciation that proceeded up to the extinction of the non-plesiosaurian sauropterygians in the Rhaetian [8]. The earliest sauropterygians recognized to date already exhibit significant aquatic specializations, such as skeletal paedomorphosis and propodial simplification and shortening [9, 10], as early as the Olenekian. The explosive radiation that followed their still enigmatic origin make Sauropterygia a model clade for successful ecological adaptation to a vacated environment that likely reflects the influence of a rapid and nearly uninterrupted aquatic adaptation and trophic optimization.

Even the oldest Lower Muschelkalk deposits of the Germanic Basin, among which is the Anisian Vossenveld Formation that crops out in the Winterswijkse Steengroeve quarry complex in the east of the Netherlands [11], already exhibit a highly diversified sauropterygian assemblage. *Nothosaurus marchicus* is among the largest recognized sauropterygian species in these deposits, smaller only than some generally rare placodonts [6, 12, 13] and undescribed Eosauropterygia known only from isolated and non-diagnostic postcranial material [6, 12, 14]. The cranial morphology of *Nothosaurus marchicus* exhibits profound dorsoventral flattening, wide orbits, large and strongly elongated temporal fenestrae, and a dentition with protruding needle-like fangs [6]. The corresponding postcranium represents that of an agile paraxial swimmer [15–17] with a propulsive bias on the anterior limbs [18]. Such observations are consistent with reconstructions of *Nothosaurus marchicus* as a piscivorous marine predator. Recent reports on the morphology and bone histology of *Nothosaurus* have improved our understanding of its secondary aquatic adaptations as well as early sauropterygian diversification in general [16, 19], but the corresponding adaptations of the neurosensory system have received only limited attention.

The modern research field of paleoneurology owes its inception to Tilly Edinger nearly 100 years ago [20]. Her first publication provided the description of a lithic endocast of *Nothosaurus mirabilis* that was obtained by sacrificing the osseous braincase [21]. The most recent endocranial exploration and associated description of several neurosensory and vascular structures of *Nothosaurus* was conducted by detailed examination of a braincase that was freed from the surrounding and enclosed matrix through acid preparation [22]. The advent of computed tomography has enabled new opportunities for paleoneurological research since the non-destructive nature of data collection permits the study of material deemed too valuable for invasive sampling [23–25]. Ongoing developments in synchrotron microtomography and particularly advances in Propagation Phase-Contrast Synchrotron X-Ray Microtomography (PPC-SRμCT) now permit virtual dissection at resolutions that are comparable to those of traditional physical sampling methods [25, 26].

The crucially different physical demands of aquatic habitats with respect to terrestrial environments are reflected in the sensory configuration of organisms that are secondarily adapted to an aquatic niche [27–29]. Studies to the sensory systems of extinct aquatic tetrapods have identified important adaptations in a variety of aquatic taxa, such as the miniaturization of the vestibular system and severe atrophy of the olfactory system in cetaceans [30, 31], and a profound reliance on vision in ichthyosaurs [32]. Recent years have seen an acceleration in the study of sensory systems in Mesozoic marine reptiles that is partially fueled by the increasing availability of non-destructive three-dimensional investigation methods. Computed tomography has revealed the morphology of the cranial endocast and endosseous labyrinth across a diverse array of taxonomic groups that had remained concealed before. Such structures are now increasingly better understood in mosasaurs [33], ichthyosaurs [34], placodonts [35], metriorhynchid crocodyliforms [36, 37], and phytosaurs [38, 39]. The neuroanatomy of early sauropterygians, however, has received only limited attention thus far [21, 22, 35, 40].

Here, we report on a description of the endocranial morphology of *Nothosaurus marchicus* based on high-resolution data acquired through PPC-SRμCT that represents the first digital visualization of a eusauropterygian cranial endocast as well as the oldest virtual sauropterygian endocast retrieved to date. This information permits an assessment of the sensory cues that *Nothosaurus* relied on during life and illustrates the adaptations that accompany the early specialization of Sauropterygia five million years after the P-T mass extinction event.

## Material and methods

**Fig 1:**
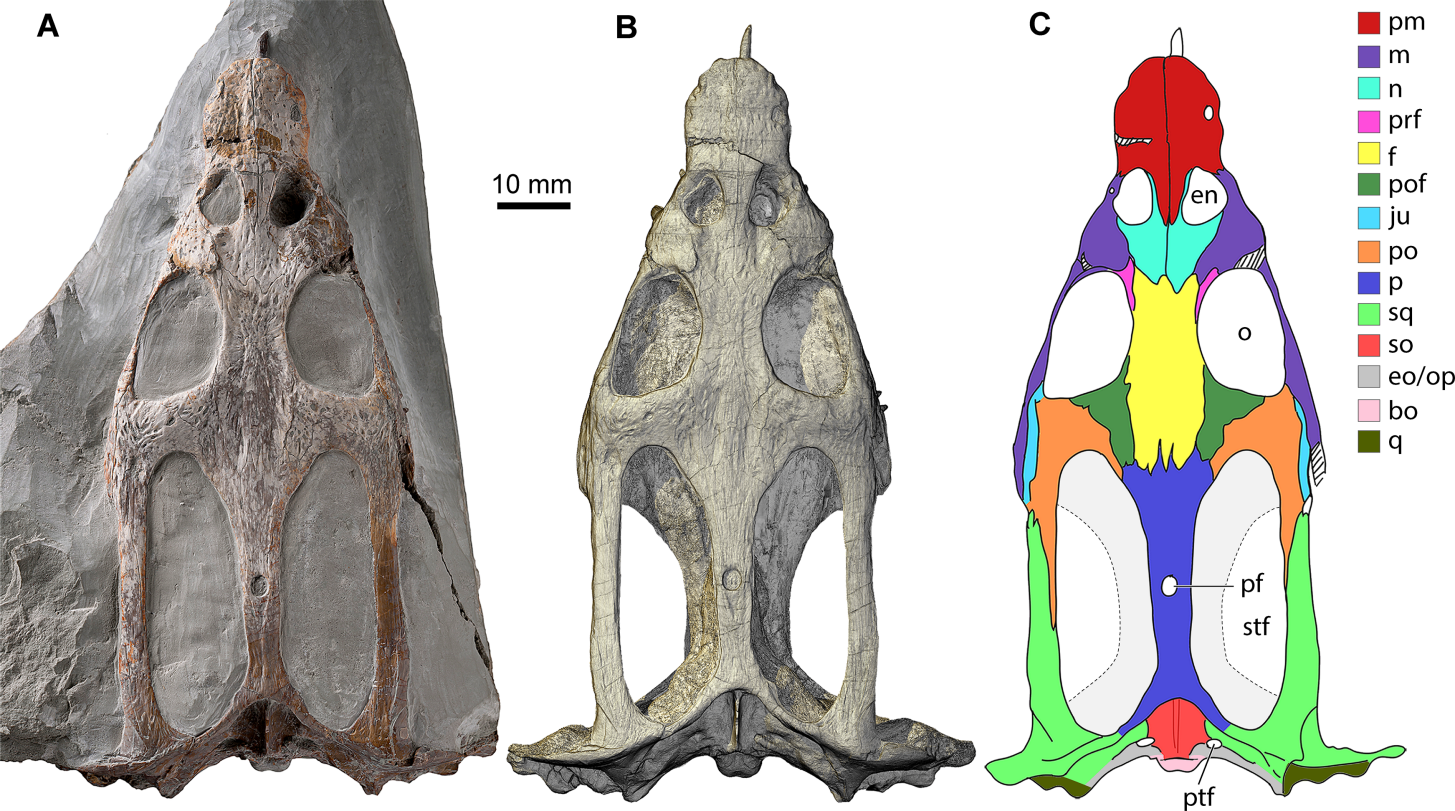
The cranium of *Nothosaurus marchicus* (TW480000375) from the Lower Muschelkalk (Anisian) of Winterswijk, The Netherlands, in dorsal view. **A.** Original cranium in matrix. **B.** Digital surface rendering. **C.** Interpretative line drawing with visible cranial bones color coded. Note that the outlines of the ventrally situated pterygoids have been indicated in light grey, bordered by a stippled line. Abbreviations: bo, basioccipital; en, external naris; eo/op, excoccipital/opisthotic; f, frontal; ju, jugal; m, maxilla; n, nasal; o, orbit; p, parietal; pf, pineal foramen; pm, premaxilla; po, postorbital; pof, postfrontal; prf, prefrontal; ptf, posttemporal foramen; q, quadrate; so, supraoccipital; sq, squamosal; stf, supratemporal fenestra.

TW480000375 (Fig 1) is housed in the collections of Museum TwentseWelle in Enschede, The Netherlands, and represents a complete cranium that has conservatively preserved its three-dimensional morphology [12, 41, 42]. Synspecific crania from this locality are known to range in length up to circa 130 mm [43]. TW480000375 has a condylobasal skull length of 101 mm and its well-ossified cranial sutures suggest that this individual had reached skeletal maturity [44]. TW480000375 was retrieved from Layer 9 (after [45]) of the Vossenveld Formation at the Winterswijkse Steengroeve locality (Winterswijk, The Netherlands). Ventral and medial aspects of the cranium remain obscured by a matrix that consists of a fine-grained micritic limestone.

### Institutional abbreviations

PGIMUH, Palaeontological and Geological Institute and Museum (now Museum für Geowissenschaften), University of Heidelberg, Germany; SMNS, Staatliches Museum für Naturkunde, Stuttgart, Germany; TW, Museum TwentseWelle Enschede, The Netherlands.

### Data accessibility

The two tomographic volumes presented and described herein are made publicly accessible as.tiff stacks through the ESRF Paleontological Database (paleo.esrf.eu).

### Visualization

TW480000375 was visualized using PPC-SRμCT conducted at beamline ID19 of the European Synchrotron Radiation Facility in Grenoble (France) to obtain sufficient contrast between the osseous cranium and the endocranial cavities and surrounding limestone matrix. Scanning data with isotropic voxel sizes of 12.82 and 28.2 μm were acquired in polychromatic mode with 13 m of propagation and at energy levels of 148 and 132 KeV, respectively. Three-dimensional volume reconstruction was conducted through filtered back projection following a phase retrieval protocol that relies on a homogeneity assumption by using a modified version [26] of the Paganin algorithm [46]. We assessed the original tomographic reconstruction as well as a recoded version of the three-dimensional data based on local texture complexity to reveal low-contrast features.

**Fig 2:**
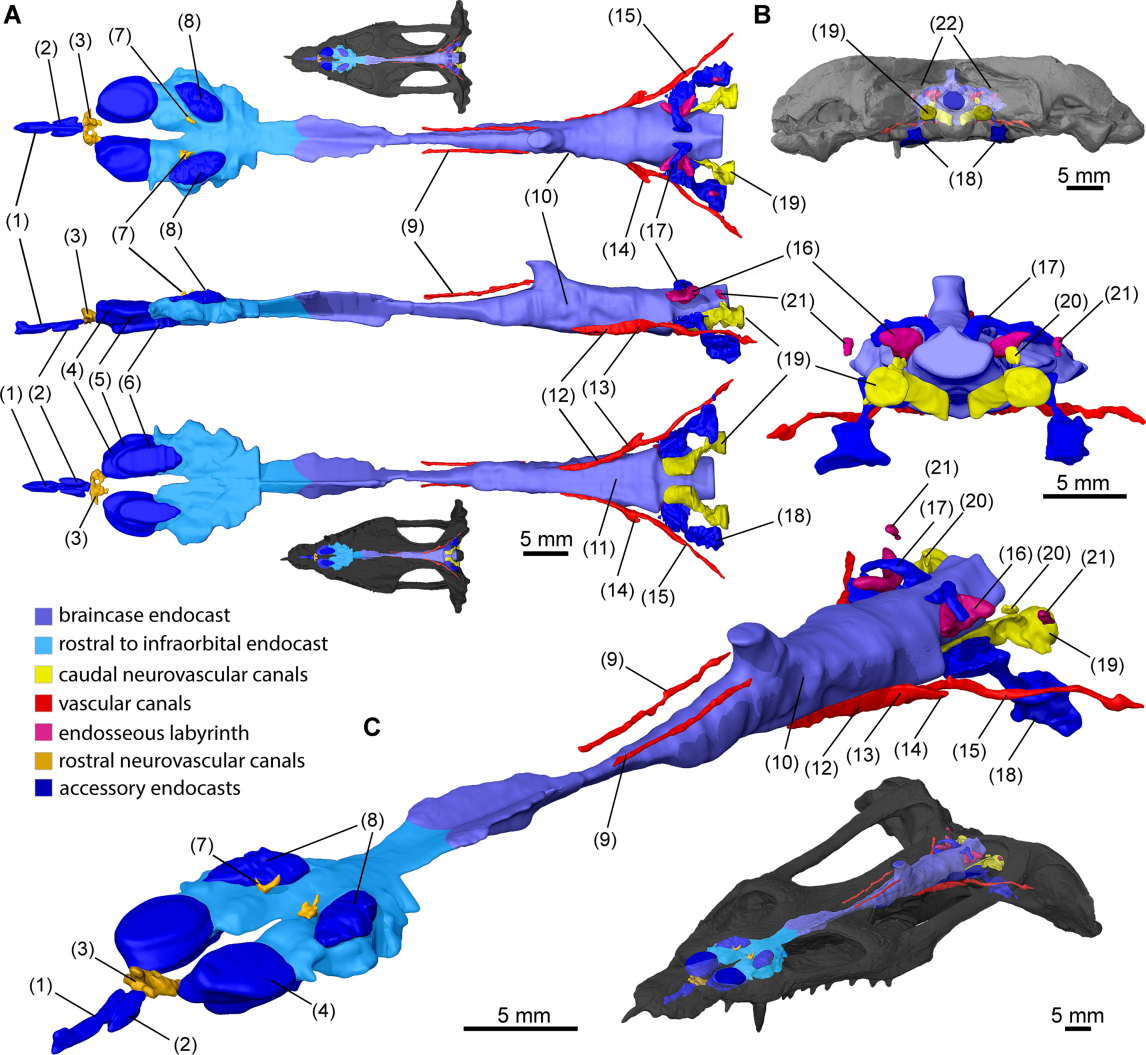
Surface renderings of cranial endocast and casts of additional endocranial voids in *Nothosaurus marchicus* (TW480000375). Different structures are color coded, integer regions excluding brain subdivisions are labeled as the structures they correlated with *in vivo*. **A.** Cranial endocast and other endocranial voids in dorsal (top), lateral (middle), and ventral (bottom) view. **B.** Cranial endocast and other endocranial voids in occipital view. **C.** Cranial endocast and other endocranial voids in angled anterodorsal view. Dorsal, ventral, occipital and anterodorsal views associated with endocast projections in cranial model. (1) premaxillary lumen; (2) vomeropremaxillary passages; (3) rostral nervous passage; (4) nasal vestibules; (5) cava nasi; (6) nasopharyngeal duct; (7) branching dorsal apertures of nasal cavity; (8) salt glands; (9) infraparietal canals; (10) endocast of cava epipterica; (11) pituitary domain; (12) interpretated location of bifurcation into sphenopalatine artery and internal carotid arch irrigating pituitary domain; (13)? cerebral branch of internal carotid artery; (14)? stapedial artery; (15) internal carotid artery; (16) apex of crus communis; (17) middle cerebral vein or paratympanic sinus; (18) interosseous casts of paracondylar interstices; (19) interosseous casts of the foramina associated with glossopharyngeal, vagoaccessory and hypoglossal cranial nerves; (20) root of hypoglossal nerve XII; (21) posterior sections of posterior semicircular canals; (22) posttemporal foramina.

**Fig 3:**
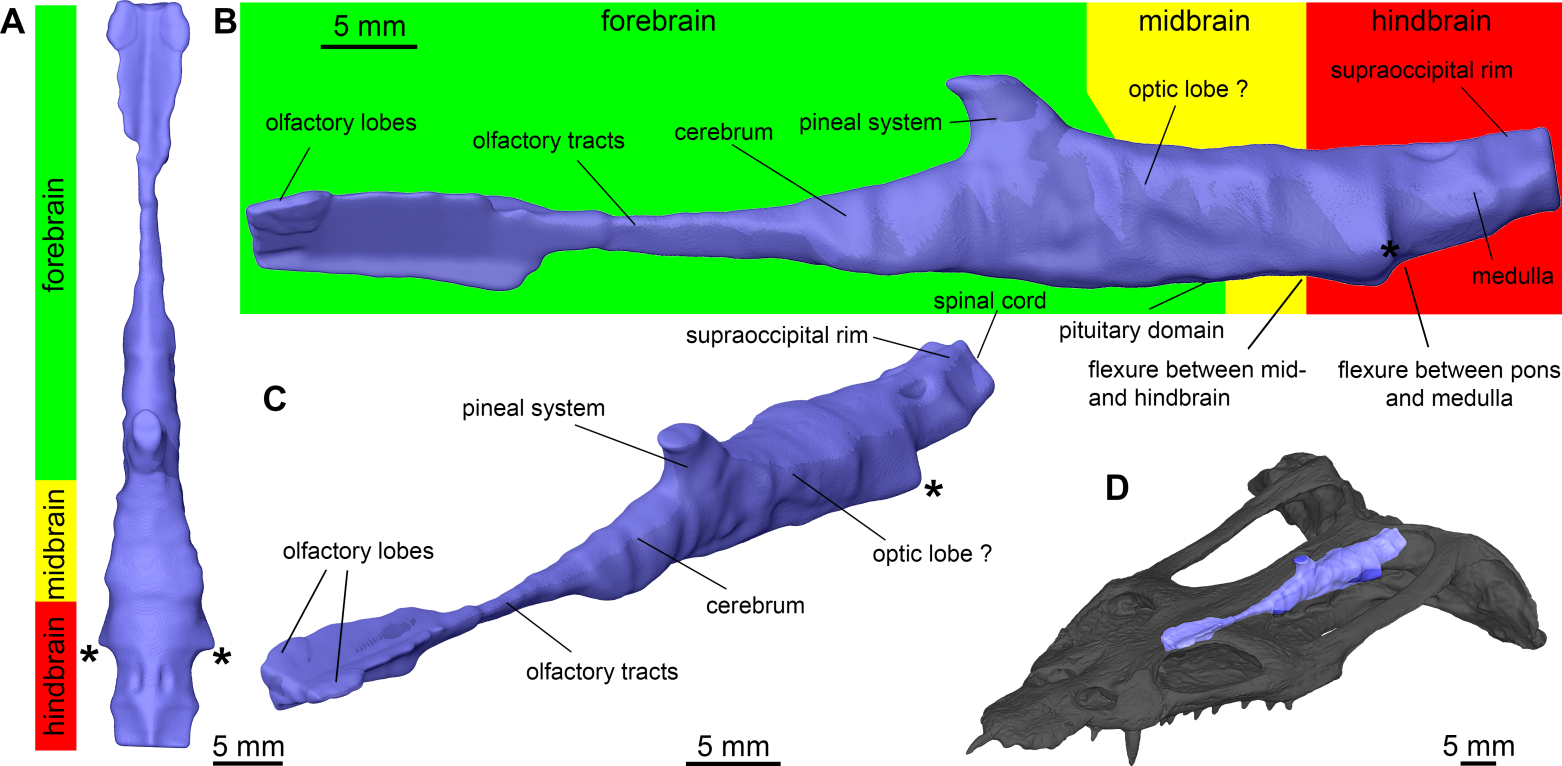
Surface rendering of the cranial endocast of *Nothosaurus marchicus* (TW480000375). Brain division and identified domains are indicated. Cranial endocast in dorsal (**A**), left lateral (**B**), and angled anterodorsal (**C**) views. **D.** Reconstructed cranial endocast projected into cranial model. Asterisks (*) indicate bilaterally positioned and transversely oriented boundaries where large hollows diverge from the central endocast.

**Fig 4:**
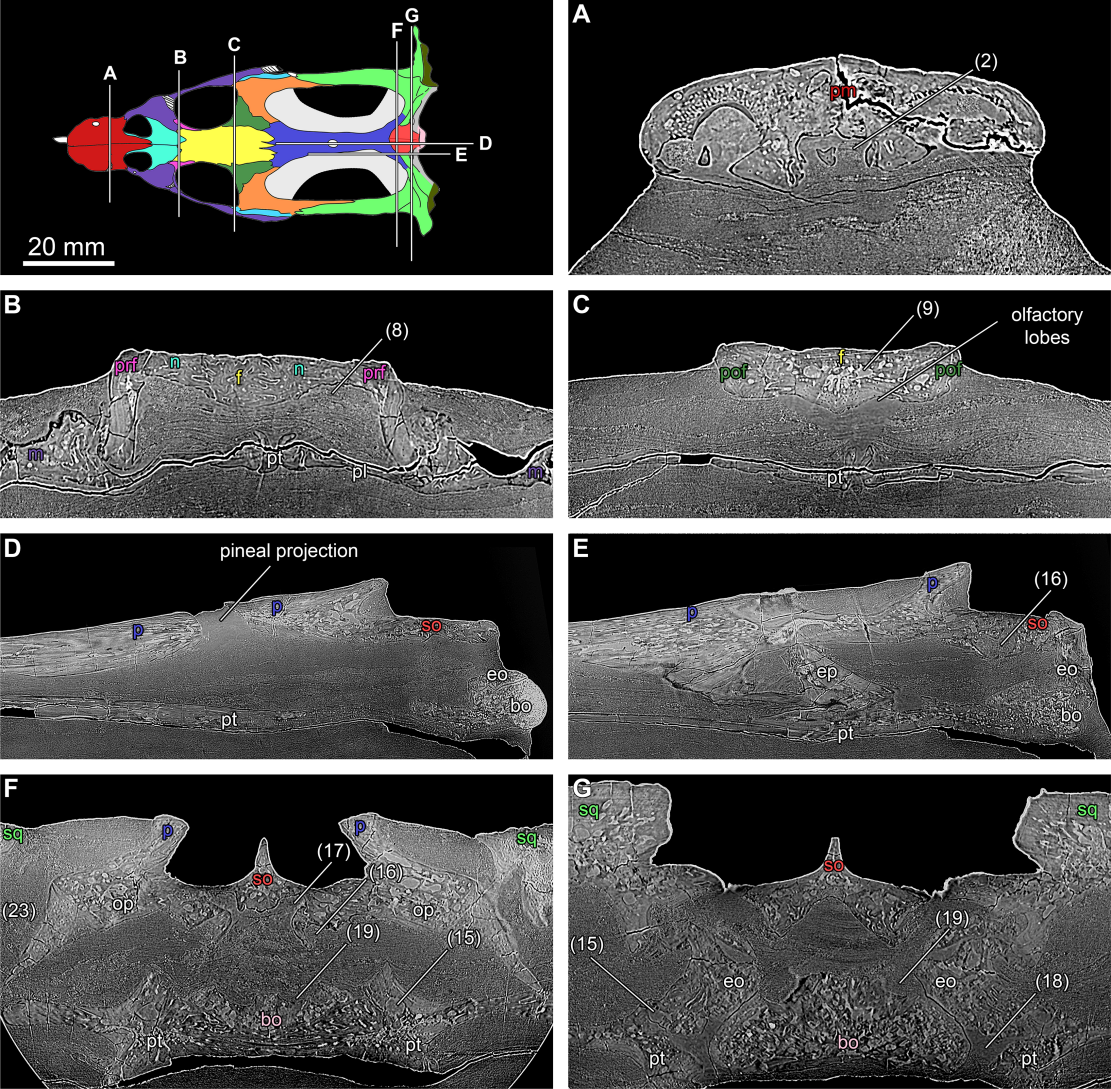
**Individual slice data (A-G) through the cranium of *Nothosaurus marchicus* (TW480000375).** Positions of the transverse (**A-C, F, G**) and longitudinal (**D, E**) sections shown in cranial surface model (see Fig 1C). Voids labeled following numbering in Fig 2. Images in A-G not to scale. Abbreviations: bo, basioccipital; eo, exoccipital; ep, epipterygoid; f, frontal; n, nasal; op, opisthotic; p, parietal; pl, palatine; pm, premaxilla; pof, postfrontal; prf, prefrontal; pt, pterygoid; so, supraoccipital; sq, squamosal.

The volumes obtained through reconstruction of the PPC-SR μCT data were segmented in VGStudio MAX 2.2 (Volume Graphics, Heidelberg, Germany) to create a virtual endocast of TW480000375 (Fig 2). The cranial endocast (Fig 3) and most of the additional morphological features reported here were extracted from the data set with a 12.82 μm voxel size (e.g. Fig 4), whereas the lateralmost domains in the posterior cranium were resolved from scan data with a 28.2 μm voxel size. The cranial endocast and additional endocranial features were rendered in VGStudio MAX 2.2 and exported at high resolution. Digital imagery and figure plates were subsequently assembled using Adobe Creative Suite 6.

### Delimitation and identification of cranial voids

Cranial topography informs on the nature and extent of the associated soft tissue anatomy and thus provides a valuable tool in defining and identifying certain intracranial domains. Since the braincase of *Nothosaurus* is well documented, we briefly recall the generic cranial osteology of *Nothosaurus* from literature [6, 22].

The ventral braincase of *Nothosaurus* is completely closed through the massive, paired pterygoids that share an interdigitated median suture and extend from the level of the central orbits back to the ventrolateral occiput. The basioccipital forms the ventromedial occiput where it defines the ventral margin of the foramen magnum and forms the occipital condyle. It is laterally flanked by the opisthotics in occipital view and dorsolaterally meets the exoccipitals that form the lateral margins of the foramen magnum. The foramen magnum is dorsally delimited by the supraoccipital that ascends anterodorsally up to the skull roof and supports a pronounced supraoccipital crest. From its posterior opening, the cranio-quadrate passage leads straight through to its anterior opening and simultaneously opens up into a medial recess between the dorsal pterygoid, ventral squamosal, and posterior prootic. Together with the opisthotic, the prootic also contributes to the otic capsule that remains medially unossified. The median basicranial floor is posteriorly formed by the dorsal surface of the basioccipital, which proceeds anteriorly onto the basisphenoid where it supports a low dorsum sellae that borders a shallow sella turcica. More anteriorly, the paired epipterygoids rise from the dorsal pterygoids up to the parietal to define laterally enclosed cava epipterica. Here, the medial pterygoids warp up to form a low ridge separating the left from the right cavum epiptericum. The roof of the braincase is largely formed by the parietal that forms a slender, anteroposteriorly elongated element appearing hourglass-shaped in dorsal view. The parietal extends along the elongated temporal fenestrae and accommodates a well-defined median pineal foramen that is placed somewhat posteriorly. Anteriorly, the pterygoid meets the frontal in a deeply interdigitating suture at a level that roughly corresponds with the anterior margin of the temporal fenestrae. The frontal extends roughly between the level of the anterior margin of the temporal fenestrae up to nearly the anterior margin of the orbits.

In *Nothosaurus*, important medial and lateral walls that bordered endocranial voids *in vivo* have not ossified [21, 22, 47], which locally prevents accurate reconstruction of such cavity boundaries during segmentation. However, a conservative approach to reconstructing the endocranium was used in these areas to link the dorsally and ventrally well-constrained parts.

Although the adult braincase of most lizards, turtles, and *Sphenodon* is not fully ossified (e.g. [48]), in archosauriforms, arguably the best studied reptilian group thus far, the brain and other intracranial structures are usually well encapsulated and thus delimited by ossified braincase elements (e.g. [37, 48–52]).

Particularly in most of the anterior parietal and frontal domain of the endocranial vault, we chose to delimit the lateral extent of the endocast along anteroposteriorly trending bone ridges extending dorsally from the pterygoids that may have supported delimiting but non-ossified structures or tissues during life. In the endocranial portions where no well-defined ridges were apparent, we adopted the ventralmost extent of the cranial roof elements and the dorsalmost extent of the cranial floor as lateral delimitations (e.g. around the frontoparietal suture). In such domains, mainly dorsal and locally ventral aspects of the endocast remain informative. Two bilaterally positioned and transversely oriented boundaries were placed where large hollows diverged from the central endocast (indicated with asterisks in Fig 3). The interpretation of soft-tissue structures is based on anatomical comparison with the cranial neurosensory systems of selected extant sauropsids, such as lepidosaurs, turtles and crocodylians, which collectively form a broad phylogenetic bracket [53] for sauropterygians.

Contrary to mammals and birds, the internal geometry of the reptilian braincase does not conservatively reflect the external geometry of the brain itself [48]. However, the close association between the dural venous sinuses and the underlying brain does allow for particular information on brain structures, such as position and relative size, to be transmitted to the internal surface of the braincase [39, 54]. Furthermore, the particularly constricted nature of the brain in *Nothosaurus* (see Discussion) ensures a more conservatively defined brain shape to be reflected in the cranial endocast than would be the case for taxa with a less constrained braincase. The location of a geometric feature on the cranial endocast allows for its identification through comparison with the phylogenetic bracket. Since the mass, and thus size, of a neural tissue is proportionally correlated with the amount of information processed during execution of the corresponding function [55], relative size comparison allows for a consideration of the importance of this function during life.

## Results

### Osteological aspects

#### Sella turcica

A dorsally projected indentation of the ventral endocast slightly anterior to the level of the pineal foramen (endocast impression: feature 11 in Fig 2) results from the contact between the basioccipital and the basisphenoid forming an incipient yet poorly preserved sella turcica on the basioccipital [22]. In TW480000375, the sella turcica is not anteriorly delimited by an anterior clinoid process but continues smoothly onto the parasphenoid. Its limited preservation does not allow for a detailed assessment of this important braincase structure, nor of the perforations known to irrigate it with blood or innervate it, as was demonstrated for the placodont sauropterygian *Placodus gigas* [35]. Well-defined projections of the hypophysis or pituitary lobes are absent on the ventral aspect of the endocast. Such an undifferentiated hypophysis also characterizes the cranial endocast of crocodylians and turtles [56–60]. The complex division of the “cavité hypophysaire” that was described by Gorce [61] cannot be recognized in TW480000375, likely due to the damage sustained in this domain. Nevertheless, the generic configuration of the sella turcica in *Nothosaurus* has been described in detail (e.g. [22]). A pituitary fossa was initially considered absent [47]. However, when it was eventually identified, its morphology was explained to have potentially accommodated an underdeveloped hypophysis relative to those of *Sphenodon* and lizards [56], where the hypophysis occupies a deep recess. In *Sphenodon*, *Varanus* and *Lacerta*, the lateral periphery of the sella turcica around the crista trabecularis serves as the origin for important oculomotor musculature, notably the *m*. *bursalis* and the retractor bulbi group [62]. The ocular muscle configuration of Crocodylia is similar to that of *Sphenodon* [63]. Furthermore, a low dorsum sellae and shallow sella turcica have been correlated with a reduction of the eyes and ocular muscles in reptiles ([64] and references therein). *Nothosaurus* exhibits a low dorsum sellae that is positioned quite posteriorly and well behind the intertemporal constriction of the braincase (see also Fig 10 in [22]). Ocular muscles attaching to the basisphenoidal region must thus have reached a considerable distance to reach the eyes (further even than the “extremely long” optic nerves [21]), including the passage of the laterally constricted section of the braincase in between the temporal fenestrae. Where considerable relative orbital size in *Nothosaurus marchicus* may indicate substantial visual acuity, this indirect pathway for ocular musculature also suggests restricted oculomotor dexterity and thus a relatively fixed ocular orientation in the cranium. However, the positioning of the orbits and relatively elongated neck in *Nothosaurus* could have granted partial binocular overlap and sufficient cervical agility (possibly even head saccades, as birds [65]) to compensate for poor intracranial ocular mobility. In other words, the constrained ocular musculature was probably compensated for by cervical mobility and a refined vestibulocollic reflex. Basal members of the genus appear to exhibit a relatively larger orbital size associated with shorter temporal fenestrae than more advanced forms that exhibit significant relative cranial elongation (e.g. [6]).

#### Cava epipterica

In the cerebral domain immediately posterior to the pineal foramen, the central to ventral aspects of the cranial endocast are laterally constricted and consequentially well defined (endocast impression: feature 10 in Fig 2) through the presence of the medially bulging epipterygoids. The ventrolateral margins of this domain are formed by distinct, anteroposteriorly trending ventral sulci that gradually even out onto the pterygoidal cranial floor anteriorly. More posteriorly, these sulci give rise to paired semitubular passages (feature 12 in Fig 2) that posterolaterally depart from the ventrolateralmost margins of this endocast domain at a small angle. The epipterygoids form the lateral walls of the cava epipterica; enclosed, paired cerebral compartments that are roofed by the palatines, floored by the pterygoids, and separated by a cartilaginous median septum *in vivo* [22]. Although the straight braincase endocast of TW480000375 exhibits a nearly flat ventral margin that prevents recognition of the transition from the forebrain to the inconspicuously expressed midbrain through a distinct flexure, the cava epipterica provides a more general indication for the position of this transition.

#### “Foramen eustachii” of Koken (1893)

The basioccipital forms the occipital condyle and the ventral margin of the foramen magnum. It meets the exoccipitals dorsolaterally along a smooth suture and shares an irregular and locally strongly interdigitating suture with the paired pterygoids ventrally. More anteriorly up to the level of the epipterygoids, the basioccipital-pterygoidal contact is deeply depressed. It continues anteriorly along the unossified lateral walls of the braincase where it gives rise to a paired, dorsally open trough (interosseous casts: feature 18 in Fig 2). More anteriorly still, these troughs pass ventral to the otic domain and join the cranial endocast at the level of the basisphenoid. Posteriorly, these troughs develop into the large, posteroventrally widening occipital gaps that result from the natural separation between the lateral basioccipital tuber and the pterygoids. These occipital corridors flanking the basioccipital have been argued to represent the passages of the eustachian tube in *Nothosaurus* [47], but have also been interpreted to (partly) accommodate the internal carotid arteries [22]. Furthermore, their variable configuration in *Nothosaurus* has been argued to indicate closure of these passages at a late ontogenetic stage [22]. In TW480000375, the trajectory of these “eustachian foramina” does not involve the internal carotids, nor does it link the pharynx with a potential middle ear cavity, as is the case for a true eustachian passage [66]. In addition, the depressed basioccipital-pterygoidal suture that distally forms the lateral troughs does not correspond to a conventional cerebral compartment but represents a dorsally unossified recess of the basicranium with an unknown function.

#### Occipital foramina associated with the glossopharyngeal, vagoaccessory and hypoglossal cranial nerves

Paired ventral troughs depart from the posteroventral braincase endocast and initially trend posterolaterally (interosseous casts: feature 19 in Fig 2). They originate as depressions of the dorsal basioccipitals and continue posteriorly between the medial margin of the opisthotics and the lateral borders of the exoccipitals. Posterior to a mediolateral constriction, these passages depart the cranium through a pair of occipital openings that flank the foramen magnum. These occipital openings are largely defined by the exoccipitals and are only laterally bordered by the opisthotics. Additionally, minute paired conical canals with a subequal diameter of 0.5–0.6 mm branch off near the posterior boundary of cranial endocast at the level of the foramen magnum (feature 20 in Fig 2). These canals project ventrolaterally where they pierce the exoccipital and join the recess of the aforementioned occipital foramina (Fig 4F and 4G). The occipital foramina between the exoccipitals and opisthotics have been explained to be homologous to the jugular foramina (metotic foramina) of extant amniotes that accommodate the pathways of the glossopharyngeal and vagoaccessory nerves [22]. Cranial nerves (CN) IX and X typically branch off closely together [56, 67] and depart the cranium through the jugular foramina in tandem with CN XI [22, 47]. The paired perforation of the exoccipital more posteriorly to the medial endocast accommodated the root of hypoglossal nerve XII that joined cranial nerves IX-XI slightly anterior to their collective cranial departure through the jugular foramina.

#### Posttemporal foramina

In TW480000375, an inconspicuous but paired perforation of the occiput resides in the sutural junction of the ventral supraoccipital, dorsolateral exoccipital and medial expansion of the opisthotic (feature 22 in Fig 2). Dorsolaterally to the foramen magnum, these concealed apertures are partially obscured by the lateral projections of the exoccipitals, rendering them nearly invisible in posterior view. They do not proceed into well-defined osseous canals anteriorly but access the non-ossified and therefore poorly resolved otic domain. Small posttemporal foramina have been identified in *Nothosaurus mirabilis* crania from the Early Ladinian of Germany and it has been proposed that reduced to obliterated posttemporal foramina are synapomorphic for Nothosauridae [22]. Their topographical position agrees with the proportionally even smaller occipital perforations recognized in TW480000375. Well-developed posttemporal fenestrae have been reported for *Placodus*, but were placed more laterally in the occiput and are delimited by the squamosal, parietal, opisthotic and possibly the pterygoid (Fig 2B in [68]). Notably, that contribution does depict potential occipital perforations of much smaller size at topographical locations that corresponds to those of the posttemporal fenestrae in *Nothosaurus* ([22]; Fig 2B in [68]). Although the posttemporal fenestrae in *Nothosaurus* are substantially smaller, they appear to be homologous to the wide apertures found in some plesiosaurs [69]. In crocodylians, the posttemporal fenestrae are small and covered over with cartilage during life [70]. The posttemporal foramina of most squamates are large vacuities, but they are completely absent in scincids [71]. No major vessel passes through the posttemporal fenestrae of extant diapsids [70, 72]. In TW480000375, they appear to communicate with the weakly defined void at or near the region that accommodated the otic capsule in TW480000375. Despite the external expression of the posttemporal openings in TW480000375, their internal morphology is inconsistent with an innervation function in *Nothosaurus*.

### Cranial endocast

#### Brain macrostructure

Overall, the osseous braincase of *Nothosaurus marchicus* is dorsoventrally constricted by the strongly flattened cranial architecture and laterally by the extremely large temporal fenestrae and associated temporal domain that accommodated the hypertrophied jaw adductor muscle complex [22]. The cranial endocast of TW480000375 consequentially takes the shape of a straight, anteriorly tapering cone, the dorsal margin of which trends anteroposteriorly (Fig 3). Its ventral margin is slightly tilted in an anterodorsal-posteroventral direction. Various additional morphological features can be resolved on the cranial endocast, which will be described and interpreted through osseous landmarks and following comparison with extant and extinct taxa.

#### Forebrain–olfactory lobes

The portion of the cranial endocast delimited by the frontal dorsally and posterior vomers and pterygoids ventrally exhibits a longitudinal, V-shaped dorsal cleft resulting from a ventrally projecting sagittal ridge on the frontal. Near the posterior orbits and lateral to the V-shaped sagittal trough, two shallow but clearly paired and distinctly ovoid recesses in the ventral frontal mark the location of the olfactory lobes (Fig 3). Slightly further posterior, a sagittal ridge dorsally terminating in a minor bifurcation rises from the interdigitating suture between the paired pterygoids. This osseous ridge extends from the midpoint between the central orbits up to the frontoparietal suture and causes a confined yet pronounced medial cleft on the ventral endocast. The subtle anteroposteriorly extending bifurcating rim carried by the sagittal upwarp of the interpteryoidal suture defines the support for a cartilaginous interorbital septum (see Fig 4C), as is expected for a tropibasic cranium [22]. During life, this septum carried the planum supraseptale; a cartilaginous trough-shaped structure occurring ventral to the frontal that forms the ventral envelope for the olfactory tracts and bulbs [21, 73–75]. The positions of the two shallow but resolvable recesses in the ventral frontal (see Fig 4C), possibly also referred to by Edinger (“between the orbits”) [21], correspond to those of the osteological correlates of the olfactory lobes in mosasaurs [76], *Varanus* [77], rhynchosaurs [78], archosaurs such as phytosaurs [73], ichthyosaurs [34], and potentially in elasmosaurid plesiosaurs [79]. This provides sufficient support for a confident recognition of the location of the olfactory lobes in TW480000375.

#### Forebrain–olfactory tracts

A medial connection extends posteriorly between the interorbital domain and the cerebral portion of the endocast (Fig 3). Since its lateral to ventrolateral margins represents artificial truncations, it only informs on the dorsal geometry of the corresponding endocranial domain. As such, it describes a gradual dorsal inflexion of the anteroposterior endocast axis at the transition from a laterally and lateroventrally poorly defined element with an incised to flattened dorsal osseous delimitation of its anterior and central domains to a more overarched and better-defined transition to the cerebral domain posteriorly. The olfactory lobes and distal part of the olfactory tracts are bilaterally divided by a sagittal ventral projection of the interfrontal suture. Posteriorly, this ventrally protruding ridge shallows until it progressively disappears, after which the passage accommodating the olfactory tracts continues posteriorly as a dorsally undivided passage [21].

#### Forebrain–cerebrum

Moving posteriorly along the confined olfactory tracts, a gradual yet conspicuous lateral divergence of the osseous walls resulting in a pronounced bilateral bulging of the endocranial void marks the anterior margin of the cerebral domain (Fig 3). The dorsal, lateral, and particularly the ventrolateral osseous margins delimiting this median void resolve bilaterally symmetrical yet faint bulges that seems to have accommodated discrete soft-tissue structures *in vivo*. The braincase appears to be constricted (e.g. [47]) by physiological constraints, which most likely resulted in a strongly sequential division of cerebral domains (see Discussion). Coupled with the presence of additional, non-cerebral tissues in the braincase vault [49], this complicates the delimitation of integer brain domains. The cerebral hemispheres, however, are readily discernible in dorsal and ventral view as a bilateral set of faint lateral bulges following the gradual widening of the olfactory tracts [21].

#### Forebrain–pineal organ

On the cranial endocast, the pineal system is expressed as an elliptic cone that is apically truncated at the pineal foramen (Fig 3). Consequentially, the pineal foramen ventrally opens up into the cranial endocast through this large, diverging and initially posteroventrally oriented passage that has an ellipsoid cross-section in the anterolateral plane. The anterior and lateral margins of this passage are subtly concave whereas its posterior margin is straight and oriented posteroventrally. More ventrally, the posterior margin of the passage exhibits an apparent deflection towards a more posteriorly trending dorsal boundary that occurs near the major body of the endocast. The walls of the pineal passage progressively diverge ventrally to meet the cranial endocast, on which it is set dorsally, in a gradual and flush manner. A well-developed, true pineal foramen in *Nothosaurus* conclusively reflects the presence of a photoreceptive pineal organ [80], also referred to as anterior parietal organ [81], which resembles the lateral eye to a remarkable degree [82]. The relatively large size of the pineal foramen and volume of the corresponding endocranial parietal passage compared to other taxa ([80]; this study) suggests a substantial reliance on the pineal complex during life for *Nothosaurus* and *Placodus* [35]. However, these voids also accommodated supportive and/or additional tissues that prevent a detailed reconstruction of the original size and morphology of the receptor system during life (e.g. [80]). Nevertheless, the relatively thick-walled parietal enclosure in TW480000375 strongly suggests that the large dorsal aperture of its pineal foramen conservatively reflects the actual size of the pineal eye, which in turn indicates a strong photoreceptive capability [80]. Although the posterior parietals of more derived *Nothosaurus* species often exhibit a pronounced lateral constriction inflicted by the enlarged temporal fenestrae, the relative size of the pineal foramen remains unchanged [83], which reinforces the conclusion of a significant dependence on the associated photosensor.

#### Midbrain and hindbrain

The posterior portion of the cranial endocast behind the cava epipterica (endocast impression: feature 10 in Fig 2) gradually widens laterally and slightly expands dorsoventrally. This expansion extends up to the level of the parietal-supraoccipital suture dorsally and extends beyond the pterygoids ventrally up to the basisphenoid-basioccipital suture. The portion of the endocast corresponding to the midbrain exhibits a subtle, apparently bilateral swelling on its dorsolateral aspect (Fig 3) that results from corresponding depressions on the ventral parietals, resembles the optic lobes identified in an ichthyosaur [34] with regard to location and orientation, and may thus represent a homologous structure in *Nothosaurus*. Although morphologically unpronounced, they do occupy one of the broadest cephalic domains, which would be consistent with a certain degree of visual acuity. Nevertheless, although extant crocodylians feature optic systems adapted for their respective niches [84], their optic lobes are poorly delimited due to the locally particularly thick dural envelope [56, 85, 86]. In crocodylians, bulges observed in this region typically reflect a portion of the venous blood system [87]. Furthermore, the lateral boundary of this endocranial domain is not continuously ossified and cannot be accurately identified throughout. These factors warrant caution when interpreting the referred features in *Nothosaurus*. At the level of the posteriormost part of the parietals, the endocast laterally communicates with paired voids that are formed by the descending flanges of the opisthotics and are excluded from the braincase endocast (Fig 4F—23). However, the referred lateral expansions in the posterior braincase endocast (asterisks in Fig 3) follow topographical contours of the ventral parietals slightly beyond the original delimitation of the brain into these voids. They thus result from artificial truncation and do not reflect the local lateral morphology of the cerebral envelope. The anterior hindbrain exhibits only a non-informative lateral expression in the domain where the floccular lobes may be expected to have resided, if present. This is comparable to the condition of extant lepidosaurian and crocodylian reptiles, which also share a small (yet distinct) flocculus [88], and disagrees with the well-developed floccular expression of birds, many other dinosaurs, and pterosaurs [89]. The floccular complex has an important role in visual stabilization and may potentially aid the reconstruction of habitual activity patterns (see Discussion, but see also [90]). Although the transition from the midbrain to the hindbrain is poorly resolved, the transition from the pons to the medulla oblongata within the hindbrain is discernable through a faint flexure and marked by dorsal and ventral “steps” in the endocast that result from a constriction associated with the parietal-supraoccipital suture (Fig 3). However, since the aforementioned artificial truncations contribute particularly to ventrolateral aspects of these “steps”, the appearance of this transition is artefactually pronounced in the endocast presented here. Between the parietal-supraoccipital constriction and the foramen magnum, the medulla oblongata tapers posteriorly, is dorsally confined by the supraoccipital and ventrally by the basioccipital, and carries a pronounced dorsal sagittal rim that extends under the (dorsal) supraoccipital crest (Fig 3).

### Sensory structures

#### Vomeropremaxillary foramina and premaxillary lumen

Two slit-like passages perforate the palate at the level of the vomeropremaxillary suture and close to the sagittal midline of the skull (feature 2 in Fig 2) to anterodorsally access a medially located lumen (feature 1 in Fig 2). This elliptic-cylindrical cavity resides completely within the premaxilla and measures circa 1.25 mm in anteroposterior diameter, 0.75 mm in width, and circa 1.30 mm in dorsoventral height. The posterodorsal margin of the lumen bifurcates into a paired and generally posteriorly trending tubular passage that can be traced up to the internarial domain (feature 3 in Fig 2). The lumen and posterior tubules connect to smaller peripheral passages that originate in surrounding rostral hollows, such as the alveoli, and at irregular grooves at the dorsal surface of the premaxillae (not depicted). An unpaired “foramen incisivum” has been previously reported for the eusauropterygian genera *Simosaurus*, *Nothosaurus*, *Cymatosaurus* and *Pistosaurus* ([91]; [7] and references therein). Conversely, a paired vomeropremaxillary foramen, such as present in TW480000375, has also been depicted in other specimens of *Nothosaurus marchicus* (Fig 11 in [42]; Figs 39 and 61 in [83]; Fig 60 in [6]; Fig 1B in [41]), as well as in *Simosaurus* (Fig 91C in [92]). Assuming this discrepancy does not represent ontogenetic disparity or a preservational, preparatory and/or visualization artefact, it illustrates that both paired and unpaired vomeropremaxillary foramina may occur within one eusauropterygian genus and thereby suggests that the contrast between singular and paired vomeropremaxillary foramina is less conservatively distributed among nothosaurian genera than presently understood. The vomeronasal fenestrae of squamates typically reside in the vomeromaxillary suture [93], whereas the antrochoanal palatal foramina of Eosauropterygia perforate the vomeropremaxillary suture. The latter condition partially agrees with that of the anterior choanae accommodating the vomeronasal ducts in *Sphenodon*, which separate the premaxillae from the anterolateral vomers [93].

We interpret that the sauropterygian antrochoanal foramina, which may be either paired or unpaired within a single genus or be absent altogether, are conservatively located in the vomeropremaxillary suture. Such foramina in plesiosaurs, when present, have been proposed to represent vomeronasal fenestrae that communicated with the Jacobson’s or vomeronasal organ [94] that appears to be plesiomorphic for Tetrapoda. Notably, the large unpaired premaxillary foramina of crocodylians exist completely within the interpremaxillary suture [92] and do not house a functional vomeronasal organ, rendering them heterologous to the palatal foramina discussed here, contra [95]. Whereas an ichthian vomeronasal system may exist in some form [96], possibly including a hybrid olfactory and vomeronasal epithelium [67], a distinct vomeronasal chemoreceptor accommodated in a nasal diverticulum makes its first appearance in non-amniote tetrapods [97–99]. Although turtles enjoy chemoreception through a vomeronasal epithelium located in the nasal cavity [100], a discrete vomeronasal system separated from the nasal cavity is commonly present in many amniotes, such as in synapsids [98] and squamates [93, 101, 102]. The tubular Jacobson’s organs of the rhynchocephalian *Sphenodon* communicate with the oral cavity through the anterior choanae [103]. They reside on the vomer and remain separated from the osseous nasal cavity only by the septomaxillae and cartilaginous elements [104]. Within extant Archosauria, discrete extra-nasal vomeronasal organs do form in the embryonic crocodylian rostrum but subsequently disappear during early development while accessory olfactory lobes never form [105]. Nevertheless, the main olfactory epithelium in the nasal cavity of *Alligator* does incorporate solitary chemosensory cells [106]. Birds lack accessory olfactory lobes and vomeronasal nerves [107], and both zebra finch and chicken genomes have been demonstrated to lack genes encoding vomeronasal receptors altogether [108].

A discrete adult amniote vomeronasal system consistently comprises three osteologically discriminable components. Those are: 1) dorsoventrally trending ducts that originate at recesses in the dorsal oral cavity, 2) a pair of lumina that typically remains separated from the more dorsolaterally residing nasal cavities, and 3) posteriorly trending vomeronasal (terminal) nerves that ultimately penetrate the cribriform plate to terminate in the accessory olfactory lobes [98, 100, 102, 109]. In TW480000375, the anteriormost palatal foramina were found to independently connect to a single, small and discrete endosseous semi-cylindrical chamber positioned anteroventrally to the narial passage. The endosseous morphology of the vomeronasal organ in *Varanus exanthematicus* [93, 110] demonstrates that the bilateral separation of its vomeronasal lumina, which are positioned directly dorsal to the vomeronasal fenestrae (Panels A-C in S1 Fig; [110]), is largely achieved through a median internasal septum [111, 112] and most ventrally through a membranous wall (see also Fig 1 in [77]). Although the vomeronasal organs in the rostrum of *Sphenodon punctatus* [93, 113] are less constrained, the corresponding topography of the narial chamber floor exhibits clear bilateral separation ([104]; Panels D-F in S1 Fig; [113]).

The single median lumen in TW480000375 lacks any morphological indication for a median partition during life. It communicates peripherally with a premaxillary infrastructure arguably homologous to the rostral canal system resolved in an Upper Jurassic pliosaur [114] and at least superficially resembling those encountered in crocodylians and some theropod dinosaurs [115]. Those structures were tentatively interpreted to represent the peripheral neurovascular plexus of a dermal sensor innervated by the trigeminal nerve [114] and may very well have facilitated a corresponding function in *Nothosaurus*. Notably, a superficially similar maxillary infrastructure was interpreted to accommodate nutritive canals in a second Upper Jurassic pliosaur [116]. Although the vomeropremaxillary domain in TW480000375 has been demonstrated to crucially deviate from the conventional tetrapodal vomeronasal architecture, the bilateral nature of the vomeropremaxillary fenestrae, ducts, and associated posterior innervation in TW480000375 lends some support for an origin through median fusion of a bilateral system. The elongated yet slender rostral morphology of nothosaurs supports a deeply rooted dentition to form a piscivorous “trapping basket” [83, 117] that may conceivably have prevented the accommodation of extensive intraosseous systems and could justify an adaptive modification of the vomeronasal system.

#### Bony labyrinth

The dorsalmost portion of the endosseous labyrinth, represented by the dorsal apex of the crus communis and the medialmost aspects of the diverging posterior and anterior semicircular canals, are preserved in TW480000375 at the dorsolateral margins of the braincase endocast posterior to the osseous constriction that defines the transition to the posteriormost braincase endocast domain (feature 16 in Fig 2). These bifurcating tubular projections arise from a descending flange of the supraoccipital lateral to the braincase endocast and the nuchal ridge. Lateral to their origin on the braincase endocast, these voids anteroposteriorly expand into a bifurcation before their abrupt lateral truncation against matrix infilling. However, if the general curvature of the posterior branches of the bifurcations is continued through the opisthotics, additional tubular sections can be discerned along this trajectory (feature 21 in Fig 2). Except for these minor tubular sections of the posterior semicircular canals, no other portions of the semicircular canals, the lagenae, and the vestibules could be reconstructed due to the absence of ossified correlates. The proximodorsal portion of the endosseous labyrinth is abruptly truncated at the level of the original sutures between (probably the epiotic contributions to) the supraoccipitals and the prootics, suggesting that both prootics were either lost or completely unossified. The anterior portion of the posterior semicircular canal and the posterior portion of the anterior semicircular canal are ellipsoidal in cross-section and measure ~0.66 mm along their major axis and ~0.45 mm along their minor axis, which renders them quite robust with respect to the size of the cranium. Furthermore, the spatial relation between the dorsal divergence of the anterior and posterior semicircular canal and the posteriormost expression of the posterior semicircular canal in the opisthotics demonstrates an anteroposterior expansion of the vestibular apparatus with respect to its dorsoventral height. This condition was also described for *Placodus* [35] and has been associated with an adaptation to an aquatic lifestyle [38, 118]. The discontinuously resolved endosseous labyrinth prevents the reconstruction of the “alert position” (see [51] for discussion).

### Respiration and osmoregulation

#### Uppermost respiratory tract

The somewhat posteroventrally tilted external nares continue posteromedially into the nasal cavity that extends between the paired nasals and the vomers. The posterior portion of the nasal cavity constricts dorsally while ventrally opening up into the oral cavity through the internal nares (choanae). These are situated behind the external nares at the junction of the vomers, maxillae and palatines, and appear to be tilted somewhat posteroventrally with respect to the palate. Slightly anterior to the posterodorsal constriction of each narial passage, the nasal is perforated by a minute and dorsally branching aperture (feature 7 in Fig 2). The dorsally bulging chambers that continue immediately ventral and posterior the external nares represent nasal vestibules (feature 4 in Fig 2) and, more posteriorly, the cava nasi (feature 5 in Fig 2). Towards the internal nares, the narial passage slightly constricts into the nasopharyngeal duct (feature 6 in Fig 2). The geometry of the narial passage between the external and the internal nares offers reduced airway resistance, which is selected upon in the narial respiratory tracts in numerous marine tetrapods [119]. We conclude that this complex represents the air passage during nasal respiration. The maxilla and vomer share a well-developed suture that separates the vomeropremaxillary foramen from the internal naris, which represents the neochoanate condition (sensu [120]) for sauropsids [93].

#### Salt glands

The lateral and ventrolateral margins of the central antorbital void are mainly defined by the paired pterygoids. Around the anterior margin of the orbits, the inconspicuous prefrontals contribute to the lateral delimitation of the medial endocranial cavity. Posterolateral to the narial passages and internal choanae, and dorsomedially and dorsolaterally confined by respectively the lateral nasals and the medial maxillae, the cast of the nasal cavities reflects the presence of two pronounced and anterolaterally—posteromedially trending oblate, bilaterally paired, and partially constrained ellipsoid recesses that align with the anteromedial margin of the orbits (feature 8 in Fig 2). They correspond with voids that are in open communication with the posterolateral narial passages but remain excluded from the posteroventral narial passages and internal nares by a low bony ridge. All extant marine diapsids require extrarenal mechanisms for salt excretion, which are universally derived from cephalic glands (e.g. [121]). This advocates the presence of cephalic salt glands in *Nothosaurus marchicus*. The developmental affinity of cephalic salt glands is variable between groups and ranges from orbital glands in sea turtles to exclusively lateral nasal glands in lizards and (accessory) sublingual glands in sea snakes, to orbital, lingual and nasal glands in crocodylians [121–127]. It has been argued that nasally derived salt glands constitute the primitive diapsid condition that arose through selection on maintaining ionic balance in particularly arid terrestrial habitats at the end of the Paleozoic [128]. The lacertid genus *Acanthodactylus* exhibits a “typical” squamate nasal architecture in which the nasal glands that reside in conchal spaces lateral to the cava nasa act as functional salt glands [126]. Fluid secreted by the lateral nasal glands passes into the nasal cavity through a secretory pore, collects in the vestibules, and is subsequently expelled by “sneezing” or allowed to dry out [126]. The paranasal recesses identified in TW480000375 are positioned posterolateral to the nasal cavity rather than purely lateral, but exhibit a similar communication with the nasal cavity as *Acanthodactylus*, which supports the inference that the paranasal recesses likely accommodated lateral nasal glands in TW480000375. The antorbital recesses in TW480000375 share their geometry, orientation and topographical position of the referred paranasal sinuses ventral to the nasomaxillary suture with the paired antorbital protuberant structures described in the marine fossil crocodylomorph *Geosaurus araucanensis* and in various other marine reptiles (e.g. [128] and references therein). Those antorbital structures have been explained to have accommodated hypertrophied nasal salt glands [128]. Such salt glands have furthermore been preliminarily reported in the Late Cretaceous polycotylid plesiosaur *Pahasapasaurus haasi* [129, 130] at a homologous location, although a detailed description has thus far been lacking. Their shared location under the medial maxillary suture posterior to the premaxillae and shared oblate spheroid geometry (indicated as “nc” in Fig 4 of [130]) corroborate the presence of homologously developed salt glands in TW480000375.

### Vascularization

#### Infraparietal canals

Pronounced anteroposteriorly trending paired tubules were encountered in the fused parietals (feature 9 in Fig 2). Each parietal contains a posteroventral perforation near its anterior margin that gives rise to a posteriorly trending tubular channel. These channels gradually verge out into the secondary tubular mesh around the pineal foramen. The dorsal head vein (vena capitis dorsalis; vcd) enters the cranial endocast posterior to the pineal foramen in *Sphenodon* [131] and is associated with the dorsal sagittal sinus in squamates [59, 72]. In the dicynodont *Niassodon mfumukasi*, the pineal foramen perforates the medial aspect of the frontoparietal suture [101], comparable to the condition of *Sphenodon* [132], whereas it occupies a medial position in the posterior part of the compound parietal in TW480000375. This disparity in osseous configuration may have originated to accommodate the extreme elongation of the nothosaurian temporal domain [83]. Slightly posterior to the frontoparietal suture in *Niassodon mfumukasi*, the local trajectories of the bilateral branches of the vcd were resolved as posteromedially trending dorsal ridges that appear to discharge the frontal anterolaterally to the pineal foramen [133]. Intrafrontal veins supplying these endocranial intervals of the vcd were not described, but may be expected to be present anterior to the pineal foramen. The aforementioned tubules in TW480000375 were encountered at a corresponding location relative to the pineal foramen in, albeit in the parietal rather than in the frontal, and verge out before actually reaching the level of the pineal foramen. As such, these intraparietal tubules in TW480000375 would roughly continue into the inferred endocranial course for the vcd of *Niassodon mfumukasi* if the endocasts were superimposed. This lends support for the interpretation that the intraparietal pathways of TW480000375 accommodated the branches of the vcd discharging the vasculature of the dorsal cranium.

#### Internal carotid branches

Paired longitudinal cylindrical passages enter the cranium from the direction of the neck through occipital foramina distinctly lateral to the foramen magnum. They subsequently penetrate the pterygoid and ultimately merge with the braincase endocast near the cava epipterica. In their trajectories, these canals gradually arc from an anteromedial course near their occipital origin to an anterior course close to their contact with the braincase endocast. A posterolateral bifurcation branches off at the level of the anterior squamosal suture with the parietal. This marks the posteromedial arrival of a second duct that lacks an osseous enclosure posteriorly to its penetration of the pterygoid. The internal carotid arteries are among the major cranial vessels in squamates [72] and crocodylians [134, 135]. The common carotid arteries depart from the dorsal branch of the aortic arch and bifurcate into an external and an internal carotid ramus before reaching the skull [72, 134, 135]. The internal carotid ramus gives rise to the cerebral carotid where the stapedial artery branches off [72, 134, 135]. After the departure of the stapedial artery, the cerebral carotid advances through the carotid canal where it eventually reaches the sella turcica. It continues anteriorly as the sphenopalatine artery [72, 134, 135]. More anteriorly still, the sphenopalatine artery runs through the vidian canal that also carries the palatine branch of the facial nerve [22, 71, 72, 136].

Most of the endocranial path of the internal carotid artery and its branches could be resolved in TW480000375, where they were found to broadly resemble the condition of *Nothosaurus mirabilis* [22]. The internal carotid penetrates the pterygoidal flange and remains entirely enclosed by bone during its lateral bypass of the hindbrain behind the cavum epiptericum in the carotid canal (feature 15 in Fig 2) and remains entirely excluded from the paracondylar interstices (feature 18 in Fig 2; see Discussion). The important connection to the sella turcica is not preserved in TW480000375, but the sphenopalatine artery that branches off simultaneously with the arch irrigating the pituitary gland must be located where the vidian canal shallows at the cavum epiptericum (feature 12 in Fig 2). Here, the corresponding artery passes onto the dorsal surface of the pterygoid [72] as it enters the basicranium at the level of the midbrain. This has previously been considered a derived condition in Nothosauroidea [22].

The encountered bifurcation may represent the departure of the stapedial artery (feature 14 in Fig 2) from the internal carotid, which would also imply that the internal carotid continues into the cerebral branch of the internal carotid (feature 13 of Fig 2) at this level. This interpretation is supported by the observations that the internal carotid artery bifurcates into the cerebral branch and the stapedial artery in the posteriormost region of the cranium in both *Iguana* [72] and *Alligator* [134]. In those taxa, the cerebral branch proceeds anteriorly to cross the sella turcica and irrigate the pituitary gland, which is partially reflected in the course of the cerebral branch in TW480000375. This is also consistent with nothosaurian material from Tunisia where the ventrolateral arrival of large carotid canals at the pituitary fossa was recorded [61]. The poorly resolved short branch of the referred bifurcation is directed posterolaterally in TW480000375, which is where the otic capsule resides. In both *Iguana* and *Alligator*, the stapedial artery proceeds dorsally towards the otic domain after its departure from the internal carotid [72, 134], where it irrigates the middle ear [100, 118]. Alternatively, this bifurcation could represent the arrival of a vein or nerve at the pterygoidal canal on its course towards the postcranium independent from the internal carotid but sharing the trans-pterygoidal osseous passage. In iguanids, both the internal jugular vein and the vagus nerve traverse the posterior cervical domain in close association with the internal carotid artery [72, 75].

#### Middle cerebral vein or paratympanic sinus

In the posteriormost segment of the braincase endocast, a bilateral pair of curved canals departs from the posterodorsal aspect of the endocranial cavity into the supraoccipitals while curving downwards and inflecting ventrolaterally into the otic capsule (feature 17 of Fig 2). These tubules arise at the suture shared by the supraoccipital and the prootic and overarch the dorsolateral bifurcation of the crus communis into the anterior and posterior semicircular canal (feature 16 of Fig 2) before opening up into the void of the otic domain ventrally. Based on comparison with extant crocodylians, the position and geometry of these passages suggest they either accommodated the middle cerebral vein [135] or allowed for communication between the paratympanic air sinus system and the paired auditory system by overarching the brain [57]. A similar structure has recently been described in a highly secondarily-adapted crocodylomorph as the dorsal dural venous sinus [36] and was recognized as unnamed cavity in a related marine crocodylomorph taxon [37]. In squamates however, this region is formed by the dorsal longitudinal sinus or the parietal sinus [72], which would be expected to have corresponded with a clear osteological correlate. These foramina are unlikely to have accommodated cranial nerves, as they arise from a dorsal region of the endocast.

## Discussion

### Comparison with earlier work by Tilly Edinger

A wealth of morphological features of the cranial endocast and the otic system of *Nothosaurus mirabilis*, the type species of *Nothosaurus*, was previously identified [21]. This cranial endocast led Tilly Edinger to recognize, for example, an enlarged pineal organ, a continuously thick medulla oblongata, an olfactory complex without hemispherical swelling, an elongated olfactory tract, and three well-developed cranial nerves; the trigeminal (V), the statoacoustic (VIII, internal opening shared with the facial nerve VII), and the vagus nerve (X, opening shared with IX). However, Edinger later recognized that her initial interpretation [21] did include several errors [137]. The unossified nature of the otic region in our specimen leads us to consider that the statoacoustic and facial nerves reported [21] were actually artefacts of the endocast. In the otic domain, she further noted the presence of the external auditory meatus leading to the middle and inner ear, the position and extent of the endocasts of the vestibular system on the lateral walls of the braincase endocast (e.g. the saccule imprints in the prootic and the utricle), as well as the horizontal semicircular canal. The other semicircular canals were reportedly damaged during preparation. The pattern of cranial ossification in nothosaurs, which will be discussed in the following section in light of heterochronic trends, argues that finer structures, such as the utricle and saccule, may also represent artefacts of the endocast. Notably, we found no trace of the horizontal semicircular canal in our specimen, which also appears to be absent in other congeneric specimens (e.g. PGIMUH K3881 and SMNS 16363, TR pers. obs.).

### Osteological aspects

#### Braincase heterochrony

The absence of (ossified) prootics suggests that TW480000375 had not yet reached skeletal maturity, which is inconsistent with the observation that the external cranial sutures appear well ossified. Prolonged or truncated physical and osteohistological maturation is a well-known trend in organisms secondarily adapted to an aquatic lifestyle [6, 9, 17, 92, 138–144]. This predominantly affects the postcranium in secondarily-adapted Mesozoic marine reptiles [9], but the specimen described here exhibits important cranial paedomorphism as well. Skeletal paedomorphosis in secondarily-adapted aquatic taxa is a common trait [144–146]. It typically results in decreased ossification during bone development, as with the extremely deferred prootic ossification in *Nothosaurus*, but it occasionally leads to deletion of cranial elements altogether [147, 148].

In TW480000375, the prootic is absent and only the occipital portion of the opisthotic is preserved. In the generalized developmental ossification sequence across reptiles, elements of the otic capsule are among the last to ossify [149, 150]. This is a common trait shared with synapsids [151], thus emphasizing the conservative nature of this pattern. The delayed, reduced or lacking ossification of components of the otic capsule is not an isolated preservational issue, as it was also observed in other nothosaurid specimens (e.g. [47, 148]; PGIMUH K3881, SMNS 16363; TR pers. obs.), as well as in plesiosaurs [139, 148]. We interpret this selective delay in ossification to result from paedomorphosis [152] through which the development of the bones forming the otic capsule was considerably delayed relative to the ancestral state of basal neodiapsids, whose osseous otic capsule is conspicuously ossified (e.g. [153, 154]).

In nothosaurids, the delayed development of bones forming and supporting parts of the braincase and the otic capsule is reflected in the absence of various structures that are commonly preserved surrounding the brain in other taxa. For example, placodonts exhibit an osteologically matured braincase that permits the extraction of several well-defined cranial nerve passages and the osseous labyrinth [35], which is also the case for the Late Cretaceous plesiosaur *Libonectes morgani* [155]. Because the lateralmost braincase walls can only be interpolated in *Nothosaurus*, definition of the topology of most cranial nerves, the vestibular organ, and several brain structures is rendered impossible. The eosauropterygian pterygoid, on the other hand, is exceptionally large [9, 156–158] and remarkably well ossified [159]. It typically extends posteriorly from the preorbital region towards its contribution to the occiput where it supports the basicranial axis bones ventrally. Although the pterygoid is one of the first bones to ossify in reptiles [149, 150], the pronounced development of the eosauropterygian pterygoid relative to the ancestral state, conversely to the condition of the otic capsule, might be the result of peramorphosis [152]. Although the postcranial anatomy of eusauropterygians appears to predominantly reflect a paedomorphic trend, the cranial anatomy seems to exhibit a mosaic of heterochronic effects. This may have important implications for phylogenetic coding, as ossification of the external cranial elements is typically considered a reliable indicator for skeletal maturity in *Nothosaurus* where alpha taxonomy is predominantly founded on cranial characters (e.g. [16, 83, 160, 161]).

#### “Foramina eustachii” of Koken (1893)

The ontogenetic plasticity [22], relatively large size, posteroventrally oriented occipital eruption, and absence of main vascular passages or potentially homologous foramina in other diapsids indicate that “foramina eustachii” probably represent taxon-specific and ontogeny-dependent morphological expressions of the junction between the basioccipital and the pterygoids rather than functional cranial foramina. Therefore, the geometry of these paracondylar interstices appears to be an architectural byproduct of the unique cranial construction of nothosauroids. Because these occipital gaps do not represent cranial foramina in the strict sense of the term, we suggest avoiding the interpretative term “eustachian foramina” and instead propose to adopt the descriptive term “paracondylar interstices” when referring to these phenomena,.

### Cranial endocast

#### Endocast macrostructure

The specialized cranial condition in *Nothosaurus* that combines dorsoventral flattening with strong lateral constriction of the braincase by the temporal fenestrae is associated with an extremely elongated linear brain morphology that exhibits an overall sequential zonation of the brain along its anteroposterior axis, including a strongly extended olfactory tract [21, 47, 56]. Although the hindbrain is broader than the forebrain, which represents the conventional reptilian condition, the forebrain and hindbrain reside at the same dorsoventral level in the unusually straight and strongly anteroposteriorly orientated cranial endocast. A generally corresponding but less pronounced linear endocranial morphology has been reported for other marine reptiles, including the closely-related placodont *Placodus gigas* [35] and a lower Jurassic ichthyosaur [34]. A similar reconstructed brain morphology of thalattosuchians [36] was recently proposed to result from the large, laterally placed orbits [85], which would have provided comparable constraints on cerebral morphology as the temporal fenestra in *Nothosaurus marchicus*.

The total volume of the endocranial cavity, excluding the poorly defined olfactory tract and olfactory lobes, amounts to circa 810 mm^3^. In reptiles, the brain itself does not fill the endocranial cavity, which in fact mirrors the external surface of the dural envelope [49]. Therefore, the brain of crocodiles and certain dinosaurs is believed to occupy only circa 50–60% of the endocranial cavity [55, 162, 163]. The relation between condylobasal skull length and full body length in *Nothosaurus marchicus*, not corrected for ontogenetic allometry, was recently described [14], and yields a reconstructed body length of circa 650 mm for TW480000375. Assuming TW480000375 exhibited a body length to body mass ratio comparable with that of *Varanus keithhornei*, its total body mass would have been circa 270 g [164]. Similarly, comparison with a juvenile alligator (610 mm [165]) yields a reconstructed body mass of 306 g for TW480000375. A corresponding brain mass (assuming a brain density of 1.036 g/cm^3^ [89]) between 0.4 (circa 50% of the braincase vault) and maximally 0.8 g (nearly 100% of the braincase vault) corresponds with a reptilian encephalization quotient (REQ; [166]) between 0.15 and 0.35. This places *Nothosaurus marchicus* within the typical range of the relation between body weight and brain weight followed by extant reptilian taxa [167].

The complex division of the brain in cerebral compartments is particularly challenging to recognize in extinct forms due to the presence of additional structures (e.g. meningeal layers) in the endocranial vault during life that usually do not preserve during fossilization [49, 56, 168]. Identification of discrete cerebral domains in fossils represented by exclusively osseous remains can only be successfully achieved through recognition and conservative application of well-understood osteological correlates of such soft-tissue structures in the cranium. For example, in the elongated brain of *Nothosaurus* with an associated sequential brain zonation, this implies that the suture between the prootic and the opisthotic, which ventrally terminates in the fenestra vestibuli, accounts for the anteriormost possible extent of the medulla oblongata, as the prootic represents an indisputable posterolateral delimitation of the cerebral domain, corroborated by a faint flexure. Particularly for taxa exhibiting a sequential zonation of the brain, the developmentally conservative relations between osseous markers and associated soft-tissue structures, such as those between the vestibular system and the cerebellum [169], are to be respected when delimiting and identifying individual brain compartments.

#### Pineal organ

Both the pineal eye and the associated pineal gland (also termed posterior parietal organ or epiphysis), which is retained as a neuroendocrine gland in numerous vertebrates lacking the pineal eye [170, 171]; see also [172]) have photosensitive capabilities [81], possibly because they represent the bilateral remnants of an originally paired structure [82, 173]. Together, these organs comprise the pineal complex [174] that, in modern lizards, influences behavior, body temperature regulation and reproductive synchrony on circadian to annual timescales through sensory stimulation of the endocrine pineal system that, for example, regulates thyroid activation [82, 170, 174–177]. Notably, contribution of the pineal eye to spatial orientation by means of a “time-compensated sun compass” when negotiating a water maze [178] and to the expression of aggressive display through an interaction between pineal endocrinal cues and thermoregulation [179] have also been documented. Finally, parietal foramen size in mosasaurs has been suggested to correlate positively with diving depth, but a hypothesized positive correlation with latitude could not be substantiated [180].

In most modern lizards, however, the size of the pineal organ strongly correlates with latitude and diurnality [181, 182], and the pineal eye may be absent altogether in equatorial species ([183] and references therein). A particular role for the pineal organ in “fine-tuning” thermoregulation in ectotherms was described and it was hypothesized that the reduction and loss of the pineal eye in Eucynodontia reflects the transition from exo- and mesothermia to endothermia [184]. Although plesiosaurs have been proposed to be homeothermic and capable of maintaining a body temperature substantially higher than that of ambient waters [185], a pineal foramen is variably present [155], and a somewhat enlarged pineal foramen was recognized in a Cretaceous plesiosaur recovered from a high paleo-latitude [186]. Pistosaurs exhibit a cortical microstructure in their long bones indicative for high growth rate and high metabolic rate [18, 138] but also retain a well-developed pineal foramen (e.g. [6]). This suggests that, in Sauropterygia, an elevated metabolic rate does not preclude the presence of a well-developed pineal foramen, as was also noted for Eucynodontia [183]. Also, the pineal organ has been described as an adaptation to terrestriality [186], which is at odds with the large size of the pineal foramen in nothosaurs.

*Nothosaurus* exhibits a cortical microstructure that indicates a moderately low growth rate [138] and suggests a comparably low metabolic rate [18, 138]. The conclusion that *Nothosaurus marchicus* relied substantially on the pineal organ (see also [80]) is consistent with an exo- to marginally mesothermic thermoregulatory strategy inferred from osteohistological proxies. *Nothosaurus* has thus far only been reported from warm epicontinental seas [138] at tropical and subtropical paleolatitudes (Western and Central Europe, Israel, Tunisia, Saudi Arabia and South China; [6]), suggesting a distribution governed by isotherms. Exothermic sea snakes, notably lacking a photoreceptor in their pineal organ [82, 177], are incapable of elevating their body temperature substantially above the ambient water temperature, even when floating at the surface. They are therefore restricted within specific surface isotherms of circa 18–20° C [187–189]. Furthermore, most sea snakes and all sea kraits are bottom foragers that rapidly equilibrate to the lower temperatures at depths up to 100 m [189]. Although *Pelamis platurus* can stand a water temperature of 5° for about an hour of time and can tolerate circa 11° C for about 36 hours, substantially longer periods endured at even 17° C are lethal [188]. Paleotemperature proxies applied to stacked Triassic marine deposits across Europe, including Lower Muschelkalk deposits from Germany, have recovered a paleotemperature range between circa 18–32° C [190]. This demonstrates that a thermoregulatory strategy similar to that of modern sea snakes (e.g. [191]) appears to have been available to *Nothosaurus* without the necessity for a photosensitive pineal organ. This, in turn, suggests that an alternative or contributing accessory function may have provided the pineal organ with a functional advantage that warranted its proportionally large size in several non-pistosauroid sauropterygians. Interestingly, the pineal organ is also known to perform a neurosecretory function governing melatonin secretion and distribution [192]. Melatonin is responsible for regulating skin pigmentation, locomotion, somnia and reproduction [193]. Melatonin controls the activity of melanophores and thereby melanin concentrations in tissues [194, 195]. Furthermore, it has been observed that the admission of pineal gland extracts to various tetrapods causes lightening of skin color [196–199]. Countershading, or Thayer's Law, describes a mode of camouflage in which an animal's coloration is darker on the upper side and lighter on the underside of the body. This pattern is found in many species of mammals, reptiles, birds, fish, and insects, both predators and prey, and has occurred in marine reptiles at least since the Cretaceous period [200]. In marine environments, countershading appears to offer camouflage particularly for mid-level dwellers towards both deeper and shallower vantage points in the water column. In a shallow marine exotherm foraging at the sea floor over a light substrate, such as *Nothosaurus marchicus*, skin coloration is intuitively subjected to the conflicting demands of promoting heat absorption while afloat at the surface (conventional countershading) and ensuring sufficient camouflage while sojourning at the sea bottom. Involvement of the remarkably large pineal foramen and potentially enlarged pineal gland could offer a functional solution for this dilemma in *Nothosaurus*. Such a hypothesis, although speculative, proposes the consideration of a mode of adaptive skin pigmentation (metachrosis) through pigment translocation that adjusts skin tone on seasonal, diurnal or even sub-diurnal timescales towards optimally balancing thermoregulation and crypsis

#### Floccular complex

Relative size of the floccular complex has been argued to proportionally reflect the requirement for image stabilization during rapid, agile locomotion in archosaurs through the vestibulocular and vestibulocollic reflexes [89], which grant a steady gaze during, for example, optical guidance while pursuing prey. This claim was recently challenged in a study assessing the relation between the size of the floccular fossa and ecology and behaviour across mammals and birds [90]. No floccular complex was recognized in TW480000375, although poor ossification of the prootic prevents reconstruction of this domain with absolute certainty. However, a floccular expression is also lacking in the endocast of the strongly ossified braincase of *Placodus gigas* [35]. Irrespective of the absolute predictive capabilities of relative floccular size and the contribution of other cerebellar regions to visual stabilization, the virtual absence of a floccular expression in the endocast of TW480000375 may be expected to correlate with reduced processing capacity and corresponding decreased performance [55], and may be linked with reduced oculomotor performance. This is intuitively more consistent with ambush predation than with endured, agile, high-speed pursuit of prey. Aquatic ambush predation guided by visual cues characterizes crocodylians (e.g. [84] and references therein), whereas vision also represents a crucial sense in short-distance ambush predation by various aquatic snakes and sea kraits [201, 202].

### Sensory systems

#### Chemoperception

While the vomeronasal organ is present in many tetrapods, only selected squamates possess a deeply cleft or forked tongue of which the tips individually communicate (either directly or indirectly through sublingual plicae [203]) with paired palatal fenestra that lead to the paired lumen accommodating sensory epithelium [101, 204]. Such an arrangement enables tropotaxis: the immediate perception of directional gradients in the concentration of certain chemical compounds after tongue flicking, which permits sampling and directional comprehension of chemical prey cues prior to attack [101, 204]. Other squamates, including ambush-hunting iguanian lizards, but also the rhynchocephalian *Sphenodon*, have a less or non-bifurcated tongue that does not permit true tropotaxis but can aid in mediating chemosensory evaluation of prey during capture by lingual pretensions or after oral contact is established [204], which does not rely on perception of a directional component. Among squamates that engage in tongue flicking, actively hunting taxa tongue-flick regularly throughout their forage, whereas ambush hunting taxa only tongue-flick when moving between ambush sites [205].

Although *N*. *marchicus* possess paired vomeropremaxillary foramina that appear homologous to the vomeronasal fenestrae of extant tetrapods, these are spaced closely together and continue dorsally into a single, shared lumen. This pattern is most consistent with the absence of a forked tongue in *Nothosaurus* and, consequently, the inability of a “squamate” mode of chemical gradient detection by means of tropotaxis. Retention of chemosensory capability in *Nothosaurus*, however, cannot be ruled out. Non-directional applications of chemoperception, such as the evaluation of potential prey, do not rely on spatial separation of sampling locations. Furthermore, in a nectic marine predator, the capability of sequentially sampling ambient water currents rather than obtaining instantaneous directional information should be sufficient to engage in tracking biochemical gradients using klinotaxis. Notably, klinotaxis represents the plesiomorphic mode of spatial chemoperception for lepidosaurs [205].

Vomeronasal chemoreception has been suggested to contribute to the array of sensory perceptions available to Sauropterygia [94]. We have observed that sauropterygian foramina incisiva occur as either one single or as two paired vomeropremaxillary foramina (e.g. [7]). Furthermore, the paired vomeropremaxillary foramina of TW480000375 communicate with a single, medially positioned lumen. The endorostral morphology of TW480000375 thereby departs from the conventional architecture of vomeronasal chemoreceptors in tetrapods but may have originated through median fusion of an initially bilaterally developed chemosensor under the morphological and ecological constraints imposed by the specialized rostral morphology of nothosaurs. It is well established that secondarily marine amniotes exhibit a reduced or even completely lost vomeronasal organ [206, 207], although a functional tropotactic vomeronasal system has been argued to have been present in mosasaurs [208]. The unique reduced endorostral morphology of TW480000375 reflects that of an atrophied vomeronasal organ with respect to those typically present in ground-dwelling tetrapods. Such an atrophied vomeronasal organ does not preclude the presence of an eusauropterygian rostral sensory system such as that proposed to enable mechano- or electrosensory perception in a pliosaur [114]. Notably, these two systems are not intrinsically linked, as chemoperception by the vomeronasal organ is achieved through innervation by the vomeronasal nerves connecting to the accessory olfactory lobes [209], whereas mechanoelectric perception is typically enabled through the facial nerve of the trigeminal nerve [210].

#### Olfaction

The limited osseous expression of the olfactory lobes on the ventral frontal prevents a confident reconstruction of their size and of the inferred neurosensory dependence on olfaction during life. Although the main olfactory system of extant marine reptiles performs poorly or not at all during submersion [100], and accommodation space for nasal olfactory epithelium may have been restricted through the presence of well-developed salt glands in *Nothosaurus* (this contribution), poorly defined osseous expressions of the olfactory lobes cannot be construed to conclusively support reduced olfactory performance. However, olfaction was evidently inhibited and will not have contributed considerably to successful subaqueous foraging. This pattern is convergent with many other secondarily aquatic tetrapods (e.g. [31, 211, 212]).

### Respiration and osmoregulation

#### Nasal cavity

A previous study argued against a primarily respiratory function for the narial passage in Eusauropterygia in general and for Nothosauria in particular [116]. Exclusive nasal respiration is inconsistent with an oral morphology supporting relatively irregularly protruding fangs that would likely have prevented a watertight seal of the oral cavity (see also [213]), for example during surfacing with a partially submerged cranium. However, in absence of a secondary palate [116], we conclude that the narial passage between the external and the internal nares did provide an air passage accessory to oral respiration, providing the choanae were not continuously covered with a palatal tissue. Size reduction of the internal nares from pistosauroids to Plesiosauria has been reported [7, 156], and the resulting size discrepancy between the external and the internal nares in plesiosaurs has been deemed a critical obstruction during respiration [119]. More importantly, the referred evolutionary trajectory is also accompanied by a gradual rearrangement of the internal nares to a location anterior to the external nares (e.g. [94, 213]), which has been proposed to coincide with the development of a functional secondary palate [116, 119]. However, more basal sauropterygian taxa exhibit the more conventional choanal placement posterior to the external nares (e.g. *Nothosaurus*) or approximately at the same level (e.g. pachypleurosaurs [214]), which is consistent with a more traditional respiratory function in these groups.

Chelonioidea accommodate a designated and strongly domed olfactory chamber (the “upper chamber” of [98]) in their nasal architecture. It has been speculated that an air bubble trapped in this dome during submersion receives volatile chemicals from water pulsed through the sinuses, which permits olfactory access to waterborne chemicals in marine turtles [100]. Compared to the more strongly domed compartments in sea turtles, the dorsoventrally flattened cranium of *Nothosaurus* only houses shallow nasal chambers in which air retention during cranial tilting or in turbulence appears highly unlikely.

#### Salt excretion

The “upper” olfactory chambers of marine Cryptodira share their topographical location and geometry with the recesses housing the postulated salt glands in *Nothosaurus marchicus* (consider the morphology of the “nasal cavity” of *Plesiochelys etalloni*; [59]). This suggests that the cryptodiran “upper olfactory chambers” and the nothosaurian antorbital recesses could represent homologous cranial voids in which glandular tissue may have exaptively replaced olfactory epithelium towards accommodating the increased need for salt excretion in the secondarily marine Sauropterygia. In extant diapsids, the secreted hypertonic solution is discharged into the nasal cavity and secreted through the nostrils [128]. In *Nothosaurus marchicus*, the postulated salt glands are contained within the posterior nasal domain and appear to remain partially separated from the narial airway by non-ossified septa, as indicated by bony ridges that supported such structures *in vivo*. Glandular discharge of hypertonic fluid likely proceeded into the cava nasa, where this solution could subsequently be expelled through the external nares, possibly aided by partial nasal respiration (as in some modern lizards [126]). Furthermore, choanal expulsion of saline discharge has been proposed specifically for Pistosauria [116]. However, since analogous modes of choanal discharge do not exist in modern taxa, phylogenetic support for this secondary mode of saline fluid emission is lacking. Additional discrete antorbital fenestra or neomorphic preorbital fenestra, such as present in *Geosaurus* and there suggested to potentially contribute to salt excretion [128], are absent in *Nothosaurus*. Lacrimal salt excretion as in sea turtles [123] appears highly unlikely for *Nothosaurus*, since sufficient interorbital accommodation space and the corresponding large interorbital foramen [214] that support lacrimal salt glands larger than the brain in sea turtles [215, 216] are absent in TW480000375.

An inferred hydrodynamically optimized “ram jet” configuration of the plesiosaurian narial passage has been proposed to benefit a speculative subaqueous operation of the main olfactory system or a vomeronasal chemosensor [213]. The main olfactory system probably does not function during submersion in extant non-chelonian reptiles [100] and we inferred that the chamber ancestrally lined with olfactory epithelium was likely at least partially occupied by the salt glands in *Nothosaurus*, which suggests a shared diminished dependence on the main olfactory system in plesiosaurs. Although the presence of hydrodynamically aided olfaction is therefore unsupported, the described forced flushing mechanism in plesiosaurs would be consistent with drainage of the hypertonic solution through the external nares rather than into the oral cavity.

### Implications for lifestyle

*Nothosaurus marchicus* is among the most common reptilian components of the faunal assemblage preserved in the coastal to shallow marine deposits of the Vossenveld Formation [12, 45]. The abundance of *Nothosaurus marchicus* relative to the comparably common but smaller-bodied pachypleurosaur *Anarosaurus heterodontus* in the paleohabitat [12, 217] would not have supported a sustainable predator-prey relationship. Furthermore, *Nothosaurus marchicus* exhibits a dental morphology unsuitable for subduing or manipulating proportionally large or particularly hard-shelled prey. Its needle-like teeth and large, recurved rostral fangs are most consistent with a principally piscivorous diet [218, 219]. *Nothosaurus* remains are mostly recovered as isolated skeletal elements interpreted to have washed up and accumulated on the tidal flats post-mortem, implying these bones form a thanatocoenosis of taxa that may not all have been preserved in the habitat occupied *in vivo* [16]. However, the degree of articulation or association of some individuals and the integrity of most material recovered [12, 16] is inconsistent with prolonged post-mortem exposure and indicates a certain proximity to the original habitats. The locally negligible gradient of the basin floor [220] therefore illustrates that *Nothosaurus marchicus* must have inhabited a shallow marine habitat, which is also corroborated by its pachyostotic ribs and limb bones [18, 19]. Such an environment should have enabled *N*. *marchicus* to traverse the entire water column to reach breathable air with ease. Notably, the combination of hyperostotic bones and piscivory in the basal cetacean *Remingtonocetus* has been argued to indicate ambush predation from a perch on the sea bottom, as rapid pursuit was deemed unlikely [221].

In the absence of indications for a pronounced development of the vomeronasal, olfactory or acoustic senses and pending the investigation of the presence and nature of integumentary mechanosensory organs in the rostrum of early sauropterygians, vision remains as the prime contactless sensory faculty. *Nothosaurus marchicus* features proportionally large orbits (~20% of total skull length), and optic lobes were potentially resolved on its endocast. It should be noted, however, that orbit size itself has been found to not absolutely correlate with eye size when phylogenetic effects are corrected for [222]. Furthermore, although no nothosauroids preserve sclerotic rings [6], various plesiosaurian specimens [69, 223–225] and European pachypleurosaurs [6] that collectively bracket *Nothosaurus* phylogenetically possess scleral rings with a relatively small internal diameter. Ichthyosaurs possessed large eyes and scleral rings with large apertures resulting in small f-numbers, which reflect adaptations to low-light environments [32, 226]. This discrepancy in scleral ring aperture between sauropterygians and ichthyosaurs may therefore also be explained by the pelagic nature and inferred deep-diving behavior of ichthyosaurs [200] that contrasts the shallow marine environments inhabited by *Nothosaurus marchicus*. Its orbital placement in the wedge-shaped cranium orients the eyes dorsally and somewhat anterolaterally, thus providing an inferred corresponding field of view with possible (partial) stereopsis. Crocodylians share this general ocular configuration and habitually engage in ambush predation, aided by their relatively low profile [227]. Modern marine predators carrying upward-directed eyes, such as particular crustaceans, typically inhabit the lower parts of the water column and use their vision to detect moving prey against the gloom above [65].

*Dikoposichnus luopingensis* is an ichnospecies reported from the Anisian of China [15] and interpreted to represent a foraging track of nothosaurid affinity. It reveals a foraging strategy that relied on punting locomotion over the seafloor envisioned to flush out crustaceans and fish that were subsequently snatched through sideway darting of the head [15]. Sojourning at or near the seafloor would have provided *Nothosaurus marchicus* with shelter against larger predators (e.g. [14]) while simultaneously enabling detection of potential overhead prey that could be ambushed and seized. As such, even the oldest known species of *Nothosaurus* already appears well adapted to a piscivorous ambush hunting strategy with an emphasized reliance on visual contrast. The intricate suite of adaptations to a marine lifestyle of late Early Triassic to early Middle Triassic sauropterygians in general and *Nothosaurus marchicus* in particular imply a profound specialization to a secondarily marine lifestyle that occurred in the first few million years after the P-T mass extinction event. Despite the more plesiomorphic appearance of Triassic sauropterygians, numerous aquatic adaptations that prefaced the prosperity of highly pelagic plesiosaurs during the Jurassic and Cretaceous were already present in the earliest such forms recognized to date.

## Conclusions

Although *Nothosaurus marchicus* from the Vossenveld Formation of Winterswijk is among the oldest representatives of the genus, its cranial architecture and corresponding endocranial neurosensory configuration involve a broad variety of cranial adaptations that may have underlain the explosive invasion of shallow marine habitats by Triassic eosauropterygians during the biotic recovery after the P-T event. Perhaps the most striking aspect of the *Nothosaurus* endocast is its simplified, straight brain morphology lacking particularly prominent cerebral portions, except for the epiphysis. Its anteroposteriorly elongated yet dorsoventrally flattened cranium with a particularly enlarged temporal musculature [22] imposed important constraints on the arrangement of neural and sensory organs and resulted in a remarkably straight brain shape. Despite these spatial limitations, TW480000375 accommodated a well-developed pineal photosensor and epiphysis, which suggests an important reliance on the corresponding pineal system *in vivo* and leads us to hypothesize may have been involved in a dynamic mode of skin tone regulation. Furthermore, vision must have presented one of the dominant distant senses, as suggested by the relatively large orbits, the potentially resolved optic lobes, and the lack of indications for other particularly well-developed remote sensory systems. The antorbital architecture of TW480000375 likely accommodated an atrophied vomeronasal organ and a rostral sensory organ, as well as well-developed salt glands, the latter two of which appear to have been retained in a Jurassic pliosaur and a Cretaceous polycotylid plesiosaur and as such may represent the plesiomorphic condition for Eusauropterygia. Specific adaptations in brain shape and certain modifications of associated organs, such as the anteroposterior elongation of the vestibular apparatus, were accommodated by heterochronic development of the cranium in general and of the braincase in particular. This differential expression of heterochronic effects warrants caution during the assessment of ontogenetic stages from cranial ossification patterns alone.

*Nothosaurus marchicus* appears to have occupied a largely piscivorous niche in shallow marine environments where a life position near the sea floor provided sufficient access to food sources through visual ambush predation, arguably some protection against apex predators, and ample access to breathable air at the surface. The spatial distribution of *Nothosaurus* between the reconstructed 18° C isotherms suggests an exothermic to possibly mesothermic thermoregulatory strategy, contrary to some Jurassic and Cretaceous pistosauroid sauropterygians.

## Supporting information

S1 Fig Vomeronasal structure in extant squamates. A-C. Virtual surface model of *Varanus exanthematicus* cranium in dorsal (A), ventral (B), and angled lateral (C) view. D-F. Virtual surface model of *Sphenodon punctatus* skull in dorsal (D), ventral (E), and angled lateral (F) view; anterior mandible excluded to reveal anterior palate in ventral view. The paired vomeronasal organ is labeled in red in both partially transparent crania. CT data sets of *Varanus exanthematicus* and *Sphenodon punctatus* were consulted on December 12 2016 through DigiMorph.org (Digimorph, 2004; The University of Texas High-Resolution X-ray CT Facility UTCT, and NSF grants IIS-0208675 and EF-0334961).
(TIF)
