# Supplementary figures and images for: Synchrotron microtomography of a Nothosaurus marchicus skull informs on nothosaurian physiology and neurosensory adaptations in early Sauropterygia
Source: PLoS One. 2018 Jan 3;13(1):e0188509. doi: 10.1371/journal.pone.0188509 (PMC5751976; doi:10.1371/journal.pone.0188509)

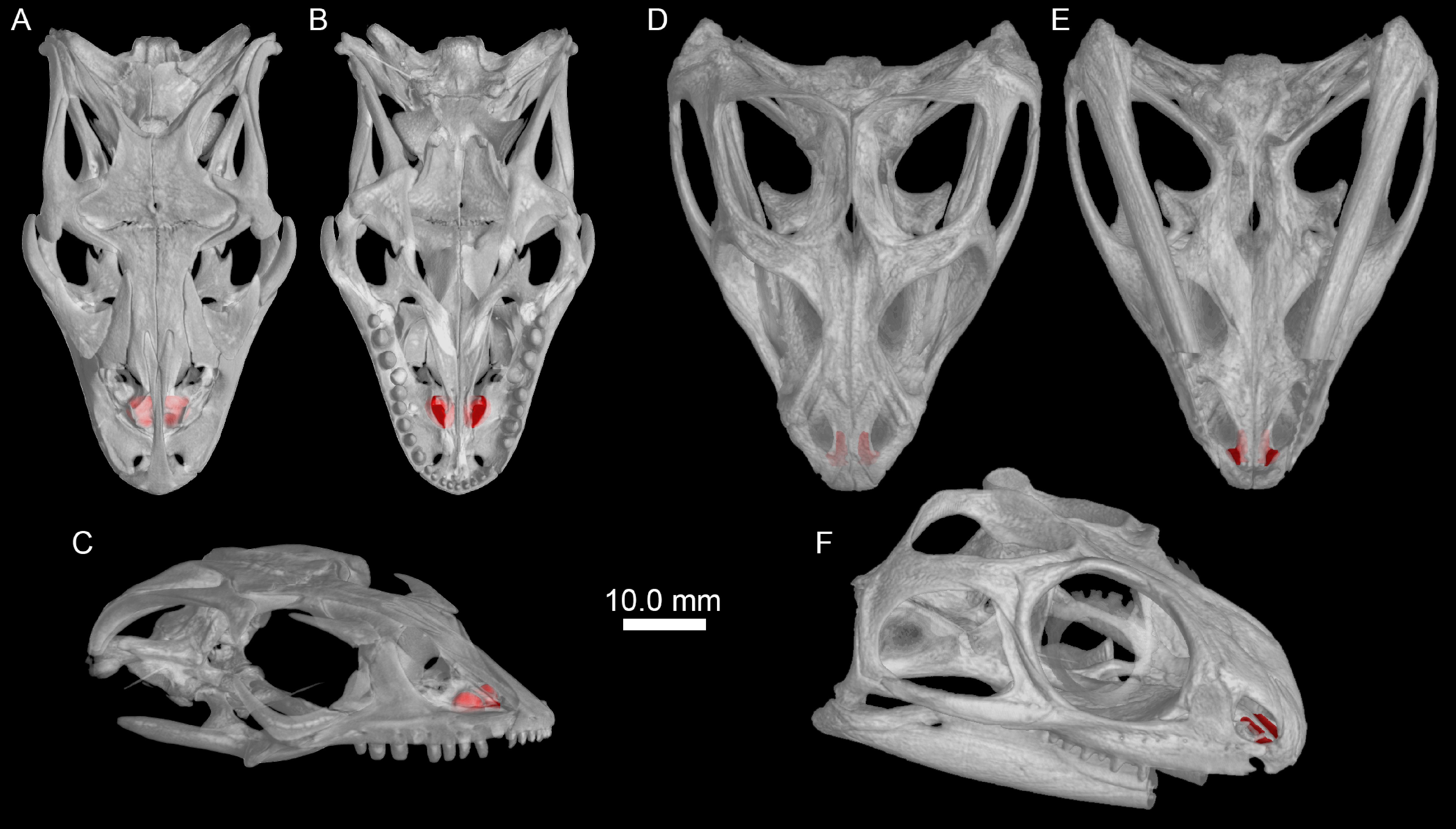

Supplement: S1 Fig — A-C. Virtual surface model of Varanus exanthematicus cranium in dorsal (A), ventral (B), and angled lateral (C) view. D-F. Virtual surface model of Sphenodon punctatus skull in dorsal (D), ventral (E), and angled lateral (F) view; anterior mandible excluded to reveal anterior palate in ventral view. The paired vomeronasal organ is labeled in red in both partially transparent crania. CT data sets of Varanus exanthematicus and Sphenodon punctatus were consulted on December 12 2016 through DigiMorph.org (Digimorph, 2004; The University of Texas High-Resolution X-ray CT Facility UTCT, and NSF grants IIS-0208675 and EF-0334961). (TIF) [file pone.0188509.s001.tif]
